# Evaluation of Gene Therapy as an Intervention Strategy to Treat Brain Injury from Stroke

**DOI:** 10.3389/fnmol.2016.00034

**Published:** 2016-05-24

**Authors:** Amanda J. Craig, Gary D. Housley

**Affiliations:** Translational Neuroscience Facility & Department of Physiology, School of Medical Sciences, University of New South Wales, SydneyNSW, Australia

**Keywords:** ischemia, viral vector, protein expression, AAV, adeno, herpes simplex virus, lentivirus

## Abstract

Stroke is a leading cause of death and disability, with a lack of treatments available to prevent cell death, regenerate damaged cells and pathways, or promote neurogenesis. The extended period of hours to weeks over which tissue damage continues to occur makes this disorder a candidate for gene therapy. This review highlights the development of gene therapy in the area of stroke, with the evolution of viral administration, in experimental stroke models, from pre-injury to clinically relevant timeframes of hours to days post-stroke. The putative therapeutic proteins being examined include anti-apoptotic, pro-survival, anti-inflammatory, and guidance proteins, targeting multiple pathways within the complex pathology, with promising results. The balance of findings from animal models suggests that gene therapy provides a viable translational platform for treatment of ischemic brain injury arising from stroke.

## Stoke: Prevalence and Treatment Options

This review considers the opportunity that gene therapy targeting neuroprotective protein expression in the brain may lend to development of novel treatments for stroke. Stroke is a leading cause of death throughout the world, and in Australia, stroke is the leading cause of severe disability; one in five people die within 1 month of their first infarct and one in three die within a year. About 88% of stroke survivors live at home and most have a disability (Banks et al., 2010; Thrift et al., 2014; Mozaffarian et al., 2015). These statistics reflect the need to develop therapeutics for stroke, whether being an ischemic event, or a hemorrhagic stroke, as there are currently limited clinical treatment options, rehabilitation often frustrates expectation, and the aging population will further exacerbate the health burden from stroke-induced brain injury.

The current treatments for acute ischemic strokes [accounting for ∼87% of strokes (Mozaffarian et al., 2015)] are the intravenous administration of recombinant tissue plasminogen activator (rtPA) to enzymatically digest the thrombi, endovascular therapy to mechanically remove the large proximal clots, or a combination of both treatment regimes, with the aim to restore blood flow to the hypoperfused area. However, the proportion of stroke patients that satisfy the criteria to undergo treatment is low. Approximately 94% of patients are ineligible for treatment with rtPA (de Los Rios la Rosa et al., 2012; Madsen et al., 2015), due to diminishing benefit and increased risk when administrating rtPA more than 4.5 h after the ischemic event, in addition to exclusion criteria which includes those patients >80 years, taking anticoagulants, with a history of previous strokes in the last 3 months, those with severe or mild strokes, or lacking a penumbral region (de Los Rios la Rosa et al., 2012; Emberson et al., 2014; Saver et al., 2015). Moreover, the effectiveness of rtPA is limited; only ∼10% of patients have a better outcome with treatment, with the site and nature of the occlusion appearing to be a factor in efficacy (Paciaroni et al., 2012; Emberson et al., 2014). Hence this approach addresses <1% of stroke incidences. There are conflicting reports of clinical outcomes following endovascular therapy, with trials indicating mechanical thrombectomy provides benefit when not coupled with rtPA, the lack of benefit of endovascular therapy with tPA, or that endovascular therapy improves patient outcomes when undertaken following tPA treatment (Broderick et al., 2013; Paciaroni et al., 2015; Saver et al., 2015). In addition to the low eligibility rate to receive treatment following acute ischemic stroke, reperfusion may result in ischemia–reperfusion injury or subsequent hemorrhage (Paciaroni et al., 2012; Emberson et al., 2014).

**FIGURE 1:**
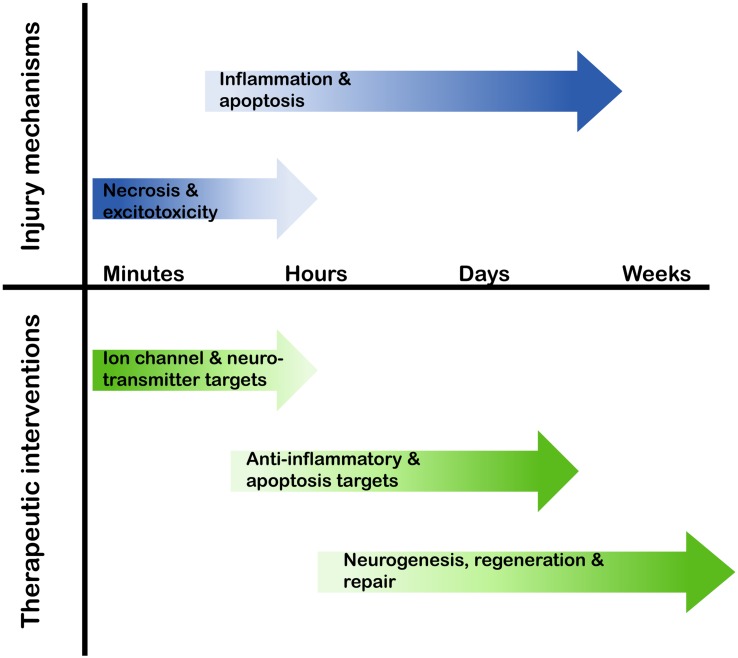
**Therapeutic target timeline following a stroke.** In the minutes to weeks following a stroke the cellular and molecular pathways that are activated alter, therefore the potential therapeutic targets and possible interventional approaches need to align with this temporal profile.

To date, there are no therapeutic interventions available to inhibit neuronal cell death, or to facilitate regeneration or neurogenesis following a neuronal injury. Research into the cellular and molecular events following an ischemic event in the brain provide a key resource for evaluation of putative therapeutics (Dirnagl et al., 1999; Moskowitz et al., 2010). Of particular interest is a range of endogenous proteins whose expression is up-regulated by stroke-induced brain ischemia, where manipulation of expression may contribute to neuroprotection, neuroregeneration, or neurogenesis. Clinically, it is essential that the manipulation of the expression profile of these proteins is matched to the therapeutic time window following stroke; for example, targeting necrosis, which occurs in the minutes following neuronal injury, may be practically unachievable, whereas manipulation of proteins that have anti-apoptotic or anti-inflammatory properties presents a far more realistic timeframe of therapy-delivery in the hours or days following a stroke (**Figure 1**).

## Considerations Around Gene Therapy Platforms

When undertaking gene therapy, in addition to the success of the treatment being dependent upon the gene target, consideration of the time of delivery in relation to the stroke onset, site of delivery, cell transduction, and onset and duration of gene expression are also critical considerations.

### Protein Synthesis

An advantage of gene delivery as a tool for administration of a therapeutic intervention in a disorder with on-going and delayed cell death is the persistence of synthesis of the therapeutic protein over a prolonged period of time (Hallek and Wendtner, 1996); thereby diminishing the need for repeated and/or frequent pharmaceutical interventions. In the context of this review, the long-term expression of therapeutic proteins has been demonstrated in rodent models of stroke (**Table 1**), with proteins evident at 7 weeks following ischemia, with an administration time-point of 3–5 weeks prior to injury, totaling 12 weeks of protein expression (Andsberg et al., 2002; Arvidsson et al., 2003).

**Table 1:** Viral gene delivery in animal models of stroke.

Protein	Viral vector	Administration (pre-/post-injury)	Stroke model	Neuroprotective	Reference
Bcl-XL	Ad	7 days pre-	Mouse; 30 min or 2 h MCAO	Yes	Kilic et al., 2002
Bcl-2	Lentivirus	3 weeks pre-	Rat; NMDA in hippocampus	Yes	Wong et al., 2005
	HSV	24 h pre-	Rat; permanent MCAO	Yes	Linnik et al., 1995
	HSV	30 mins post-	Rat; 1 h MCAO	Yes	Lawrence et al., 1997
	HSV	1.5 h post-	Rat; 1 h MCAO	Yes	Yenari et al., 2001
	HSV	4 h post-	Rat; 1 h MCAO	No	Lawrence et al., 1997
Bcl-w	rAAV	3 weeks pre-	Rat; 1.5 h MCAO	Yes	Sun et al., 2003
BDNF	rAAV	4–5 weeks pre-	Rat; 30 min MCAO	Yes	Andsberg et al., 2002
	rAAV	14 days pre-	Rat; various MCAO models	Yes	Zhang et al., 2011
CDNF	rAAV	2 days post-	Rat; 1 h bilateral CCA and right MCAO	No	Matlik et al., 2014
CNTF	Ad	7 days pre-	Mouse; 30 min MCAO	Yes	Hermann et al., 2001b
GDNF	Lentivirus	3 weeks pre-	Rat NMDA to hippocampus	Yes	Wong et al., 2005
	Ad	7 days pre-	Mouse; 30 min MCAO	Yes	Hermann et al., 2001a,b
	HSV	4 days pre- and	Rat; 1 h MCAO	Yes	Harvey et al., 2003
	HSV	3 days post-	Rat; 1 h MCAO	No, but behavioral improvement	Harvey et al., 2003
	Ad	During and	Rat; 1.5 h MCAO	Yes	Zhang et al., 2002
	Ad	1 h post-	Rat; 1.5 h MCAO	No	Zhang et al., 2002
	rAAV	During	Rat; 1.5 h bilateral CCA and right MCAO	Yes	Tsai et al., 2000, 2006
HB-EGF	rAAV	6–7 days post-	Rat; 80 min MCAO	No, but functional recovery with neurogenesis and angiogenesis	Sugiura et al., 2005
NGF	rAAV	4–5 weeks pre-	Rat; 30 min MCAO	Yes	Andsberg et al., 2002
NT3	rAAV	24 h post-	Rat; endothelin-1	No, but improved behavioral and sensory outcomes	Duricki et al., 2016
HSP-27	HSV	3 days pre-	Rat; 30 min MCAO	Yes	Badin et al., 2006
	HSV	30 mins post-	Rat; 30 min MCAO	Yes	Badin et al., 2009
HSP-70	HSV	3 days pre-	Rat; 30 min MCAO	No	Badin et al., 2006
	HSV	30 mins post-	Rat; 30 min MCAO	No	Badin et al., 2009
HSP-72	HSV	24 h pre-	Rat; 1 h MCAO	Yes	Yenari et al., 1998
	HSV	17 h pre-	Rat; 8 min bilateral CCA	Yes	Kelly et al., 2002
	HSV	0.5 and 2 h post-	Rat; 1 h MCAO	Yes	Hoehn et al., 2001
	HSV	5 h post-	Rat; 1 h MCAO	No	Hoehn et al., 2001
Gpx	HSV	12 h pre-	Rat; 1 h MCAO	Yes	Hoehn et al., 2003
		2 and 5 h post-	Rat; 1 h MCAO	Yes	Hoehn et al., 2003
CXCL12 (SDF-1α)	Ad	3 days pre- and	Rat; 1.5 h MCAO	Yes	Yoo et al., 2012
	Ad	7 days post-	Rat; 1.5 h MCAO	Yes	Yoo et al., 2012
	rAAV	7 days post-	Mouse; permanent MCAO	Protects myelin sheath	Li et al., 2015
	rAAV	7 days post -	Mouse; permanent MCAO	Yes	Li et al., 2014
IL-1 receptor antagonist	rAAV	During	Rat; 1.5 h bilateral CCA and right MCAO	Yes	Tsai et al., 2003
Netrin-1	rAAV	1 day post-	Rat; 1 h bilateral CCA and left MCAO	No, but increased vascularisation and improved behavior	Sun et al., 2011


*The efficacy of gene therapy as a treatment option following stroke, to facilitate the expression of various proteins, has been assessed in several animal models of stroke with the administration of viral vectors tested both pre- and post-ischemia. Outcomes of neuroprotection varied between infarct volume and neuronal cell counts. Ad, adenovirus; rAAV, recombinant adeno-associated virus; BDNF, brain derived neurotrophic factor; CCA, common carotid artery; CDNF, cerebral dopamine neurotrophic factor; CNTF, ciliary neurotrophic factor; CXCL – CXC chemokine ligand; GDNF, glial cell-derived neurotrophic factor; Gpx, glutathione peroxidase; HB-EGF, heparin-binding epidermal growth factor-like growth factor; HSP, heat shock protein; HSV, herpes simplex virus; MCAO, middle cerebral artery occlusion; NMDA, N-methyl D-aspartate; NT, neurotrophin; SDF, stromal cell-derived factor.*

Conversely, the reliance of host-mediated protein synthesis of the viral-encoded sequences, following an ischemic event, may be compromised, and, therefore, result in diminished putative therapeutic efficacy, due to the inherent reduction in protein synthesis associated with the brain injury (Kleihues and Hossmann, 1971). Specific to gene delivery, Lawrence et al. (1997) demonstrated herpes simplex virus (HSV)-mediated expression increased at 12 h following ischemia in ischemic tissue; however non-ischemic tissue had increased expression, to a greater extent, as early as 8 h post-ischemia. These data not only highlight a delay in peak protein expression, but also a reduction in the extent of expression with the ischemic brain injury (Lawrence et al., 1997).

### Delivery Site

Down-regulation of protein synthesis can be overcome, in part, by viral delivery into a non-affected region of the brain, or to the peri-infarct area, as opposed to the ischemic area (Zhao et al., 2003; Matlik et al., 2014). This approach not only overcomes potential synthesis suppression, but delivery to striatal peri-infarct regions of the brain, post-ischemia, has shown to be an effective means of viral distribution, with viral particles hypothesized to travel toward white matter tracts during the period of edema (Matlik et al., 2014). There is also evidence of virally derived proteins in brain regions remote of the initial viral delivery site (Hermann et al., 2001b). Alternatively, viral delivery into sites remote of the brain, such as a stroke-affected limb may promote corticospinal axonal sprouting in the spinal cord from the less affected hemisphere driven by the viral expression of neurotrophin-3 (Duricki et al., 2016). This could present as an alternative approach to the problem of inhibition of axonal re-growth in areas with astrocytic scarring. In contrast, studies have also shown increased striatal neuronal loss following post-ischemia anterograde delivery of GDNF. Whether this result is due to the delivery mode, the protein being expressed, the relative time of expression, or a combination of all of these, as well as additional factors is yet to be determined (Arvidsson et al., 2003).

An alternative approach to overcome the time-delay of protein expression is to express the viral-derived proteins in stem cells, which are then transplanted by intracerebroventricular injection (Watanabe et al., 2004; Chen et al., 2016). This method has resulted in reduced infarct volume and increased behavioral outcomes and may be a viable adjunct to gene delivery, with clinical trials of stem cell therapy in stroke patients already well established (Jeong et al., 2014).

### Viral Vectors

The design of the viral vector is an important component in gene delivery, ensuring that the virus is not pathogenic or induces neurotoxicity, targeted cell-specific delivery can be facilitated or alternatively the vector can be developed for broad transduction, and gene expression duration can be appropriately modulated. The desired expression profile of the protein should be considered in terms of expression instigation, duration, and efficacy. The four most commonly used viral vectors are HSV type-1 (Bloom et al., 1995; Carpenter and Stevens, 1996), adenovirus (Ad; Akli et al., 1993), recombinant adeno-associated virus (rAAV; Hallek and Wendtner, 1996), and lentivirus (Naldini et al., 1996). Each have their own innate attributes and deficiencies, which must be considered in relation to the size of the gene sequence to be inserted, the target cell population, and the protein expression profile. In addition to the innate variations between viral vector system, viral serotypes will also affect the target cell specificity and protein expression (Davidson et al., 2000; von Jonquieres et al., 2013, 2016). As noted below, HSV vector-mediated expression has been reported with a few hours (Hoehn et al., 2003), whereas other commonly used viral vectors exhibit expression profiles that take days or weeks to establish (Mason et al., 2010). Further alterations in the expression profile of the protein of interest can be driven with capsid modifications, as well as the promoter used to drive gene expression, which can bias glial versus neuronal expression, and the potential to incorporate gene cassette control elements (von Jonquieres et al., 2013, 2016). The broad consideration of technical development of gene therapy platforms for clinical applications, including non-viral modalities, and use of gene regulatory strategies such as shRNA are outside of the scope of this review, which is a perspective on the opportunity and exemplar prospective gene targets.

## Therapeutic Protein Candidates for Stroke Treatments

### Bcl-2 Family

In terms of a gene-delivered therapy following stroke, the anti-apoptotic proteins within the B-cell lymphoma-2 (Bcl-2) family, including Bcl-2 itself, Bcl-extra long (Bcl-XL), and Bcl-2-like 2 (Bcl-2l2 or Bcl-w), are an obvious therapeutic choice due to their intrinsic role in modulating apoptosis and neurogenesis (Czabotar et al., 2014). Evidence of the neuroprotective capabilities of Bcl-2 has been demonstrated in a variety of injury models, with roles including modulation of intracellular Ca^2+^ concentration (Zhong et al., 1993; Murphy and Fiskum, 1999), reducing reactive oxygen species (Kane et al., 1993), and inhibiting the translocation of apoptosis-inducing factor (Zhao et al., 2004), all of which are prevalent following stroke. Furthermore, transgenic mice experiments have shown that over-expression of Bcl-2 provides neuroprotection following ischemia (Kitagawa et al., 1998). In addition, the over-expression of Bcl-2 induces neurogenesis following ischemic injury (Lei et al., 2012).

The therapeutic effectiveness of Bcl-2 anti-apoptotic proteins, expressed from viral vectors including HSV (Linnik et al., 1995; Lawrence et al., 1997; Yenari et al., 2001), Ad (Kilic et al., 2002), rAAV (Sun et al., 2003), and lentivirus (Wong et al., 2005) has been demonstrated in middle cerebral artery occlusion (MCAO) and bilateral common carotid artery (CCA) occlusion models of stroke in rodents, as well as a model of excitotoxicity (**Table 1**). The administration of the viral vector ranged from 3 weeks pre-ischemic insult to 4 h post-ischemia, which provide proof-of-principle data, but are sub-optimal for clinical translation. The most promising studies utilized administration of a HSV-Bcl-2 construct at 30 and 90 min following MCAO, with significant neuroprotection achieved (Lawrence et al., 1997; Yenari et al., 2001). Disappointingly, there was a lack of neuroprotection afforded at the 4 h post-ischemic administration time-point, which is postulated to be due to decreased protein synthesis following ischemia (Lawrence et al., 1997).

### Heat Shock Proteins

The heat shock proteins (HSP) are stress-related proteins with chaperone properties. Of particular interest are the HSP-70 family, comprising of the constitutive HSP-70 and the homologous inducible HSP-72, which are up-regulated following cerebral ischemia (Brea et al., 2015). The over-expression of HSP-72 proteins in transgenic mice has provided evidence of the neuroprotective role following cerebral ischemia (Xu et al., 2011). In addition, the smaller HSP-27 (also known as HSP-25), similarly, provides neuroprotection following ischemia, when over-expressed in a transgenic mouse model (van der Weerd et al., 2010). The success of the HSPs in providing neuroprotection when transduced has been varied (**Table 1**), with studies finding neuroprotection evident with HSV-HSP-72 delivery 3 days pre-insult to 2 h post-insult, but not when administered 5 h post-ischemia (Yenari et al., 1998; Hoehn et al., 2001; Kelly et al., 2002). Conversely, HSP-70 did not confer neuroprotection when administered 3 days pre- or 30 min post-ischemia, while HSP-27 did with similar administration and injury models (Badin et al., 2006, 2009). These differences may, in part, be due to the method in which neuroprotection is measured, with studies varying from counts of transduced surviving striatal neurons to infarct volume analysis following magnetic resonance imaging (Kelly et al., 2002; Badin et al., 2009). Additionally, the neuroprotective effect of HSP-72 may lie not only with its innate role in protein chaperoning, but also in the induction of Bcl-2 expression, possibly enhancing the neuroprotective effect following ischemia (Lawrence et al., 1997).

### Antioxidant Enzymes

Antioxidant enzymes, such as superoxide dismutase (SOD), catalase, and glutathione peroxidase (Gpx), are postulated to reduce brain damage incurred due to increases in reactive oxygen species following stroke. Transgenic animal studies have shown that over-expression, or deficiencies, of antioxidant enzymes affects the outcome following stroke (Murakami et al., 1997; Chan et al., 1998; Kawase et al., 1999). Gene delivery of Gpx both pre- and up to 5 h post-MCAO conferred neuroprotection, in conjunction with an increase in Bcl-2 (**Table 1**). It is proposed that the neuroprotective effect seen with administering the gene therapy at 5 h post-ischemia may be attributed to both the benefit of the antioxidant action, as well as the anti-apoptotic properties of Bcl-2, accounting in part for why the Bcl-2 administration alone was not neuroprotective when administered 4 h post-ischemia (Hoehn et al., 2001). The HSV construct was reported to drive Gpx expression at 4–6 h post-administration, which indicates the therapeutic time window for the Gpx action was 9–11 h post-MCAO, in the rat model. This is in line with the belief that complex pathologies such as stroke will require therapeutic agents to target multiple pathways for inhibition and/or activation to be truly efficacious (Moretti et al., 2015).

### Neurotrophins

Neurotrophins have a role in the regulation of neuronal tissue development and repair, promoting survival, differentiation, and maintenance in physiological and pathological conditions. Neurotrophin gene cassettes, therefore, offer broad potential for therapy following stroke (Lindholm et al., 2007; Machalinski, 2014; Otsuka et al., 2016). Experimentally, various *in vitro* and *in vivo* ischemic injury models have been utilized to demonstrate the neuroprotective efficacy of neurotrophins, including brain-derived neurotrophic factor (BDNF; Zhang and Pardridge, 2001; Otsuka et al., 2016), glial cell-derived neurotrophic factor (GDNF; Yuan et al., 2013), and nerve growth factor (NGF; Semkova and Krieglstein, 1999; Tabakman et al., 2005). This has been further translated to delivery of gene cassettes for the recombinant neurotrophic factors, including BDNF (Andsberg et al., 2002; Zhang et al., 2011), GDNF (Tsai et al., 2000, 2006; Hermann et al., 2001a,b; Zhang et al., 2002; Harvey et al., 2003; Wong et al., 2005), ciliary neurotrophic factor (CNTF; Hermann et al., 2001a), and cerebral dopamine neurotrophic factor (CDNF; Matlik et al., 2014). These gene therapy agents have been shown to provide neuroprotection with viral vector delivery, including HSV, Ad, and rAAV, in MCAO models of stroke in rodents (**Table 1**). When the viral vectors were administered either pre-ischemia, during, or up to 1 h following ischemia, the infarct volume was significantly reduced, as was caspase-3 expression (Hermann et al., 2001a,b; Zhang et al., 2002; Harvey et al., 2003; Matlik et al., 2014). Further increasing the therapeutic window to 6–7 days post-injury, with the administration of heparin-binding epidermal growth factor-like growth factor (HB-EGF; Sugiura et al., 2005), did not provide the same reduction in infarct volume. This outcome may be expected considering the timeline of neuropathological pathways activated in relation to therapy administration time. However, there was an increase in angiogenesis and improved functional recovery, modulated by the gene delivery of HB-EGF. As clinical outcomes in humans are not measured in terms of infarct volumes but rather as an improvement of motor function and cognition, these results in animal models are encouraging for translation of the gene targets, demonstrating a positive outcome coupled with a clinically relevant administration timeframe. Therapeutic targets for the neurotrophin signaling cascade may be very broad, including the neurotrophins themselves, the corresponding tropomyosin-related kinase (Trk) receptors (also referred to as receptor tyrosine kinases), and potentially second messenger-coupled effectors such as ion channels modulated downstream of phospholipase Cγ activation.

### Chemokines

Chemokines are inflammatory mediators that are up-regulated following stroke, with a role in recruiting leukocytes to the area of damage in the brain. The resulting inflammation in the brain can increase the severity of the stroke, or conversely the recruited phagocytes aid in cellular debris clearance, or a combination of both (García-Berrocoso et al., 2014). The expression of the chemokine CXCL-12, otherwise known as stromal cell-derived factor-1 (SDF-1), is constitutively expressed in the brain, with increased expression occurring following ischemia (Wang et al., 2012). Studies modulating endogenous CXCL-12 following stroke provide contrasting results. The inhibition of CXCL-12 with the receptor CXCR4, by delivery of receptor antagonist during the acute post-ischemic time period, improved behavioral recovery and reduced infarct volumes (Ruscher et al., 2013). Similarly, rAAV gene delivery of the IL-1 receptor antagonist reduced infarct volume (**Table 1**) (Tsai et al., 2003). However, in a study with forced limb use following stroke, the administration of the CXCR4 antagonist resulted in a deficit in recovery with worse motor and cognitive outcomes (Zhao et al., 2015). Gene delivery studies have provided evidence of the benefit of CXCL-12 following stroke. Adenoviral or rAAV gene delivery of CXCL-12 into mice and rats, administered from 3 days pre-ischemia to 7 days post-ischemia reduced brain atrophy, maintained myelin sheath integrity, increased oligodendrocyte progenitor cell proliferation and migration, and the promotion of angiogenesis (Yoo et al., 2012; Li et al., 2014, 2015). Further contrast is seen in clinical studies, with a positive correlation between increased serum CXCL-12 levels and poor outcome in stroke patients in a Chinese cohort, but increases in CXCL-12 serum levels in patients transplanted with autologous mesenchymal stem cells correlating to improved outcome (Lee et al., 2010; Liu et al., 2015; Cheng et al., 2016).

### Guidance Proteins

In contrast to therapeutic proteins targeting neuroprotection, gene therapy can also be utilized to express proteins to aid in regeneration. Netrins are axon and cell guidance proteins, that are expressed both during neural development and in the mature nervous system in physiological conditions, with up-regulation of expression occurring in the peri-infarct region 14 days following ischemia (Moore et al., 2007; Tsuchiya et al., 2007). Gene delivery of netrin-1 with rAAV, 1 day following ischemia, resulted in an increase in peri-infarct vascularisation and immature neuronal migration. Despite the lack in reduction of infarct volume, there was an improvement in post-stroke locomotor activity, motor asymmetry, and exploratory behavior (Sun et al., 2011).

## Conclusion

Stroke is a complex pathology with a multitude of biochemical, cellular, and molecular pathways instigated differentially over time, providing a challenge to target therapeutically, while also providing multiple opportunities for intervention. The aim for stroke researchers to develop therapeutics that will increase survival probability of patients, as well as improve cognitive and behavioral recovery, whilst ensuring therapeutic delivery within a clinically relevant timeframe is challenging. However, enhancing the expression of endogenous proteins or facilitating expression in areas most susceptible to damage, by gene delivery, provides promise, with progress being made in both the therapeutic window for delivery and an expanding range of potential protein targets. The use of these therapies in conjunction with the currently available treatments, such as rtPA or mechanical clot removal, is an additional area of research to be explored. Despite the promising progress, further research will need to be undertaken before these therapies reach clinical trials, as the regulatory challenges for gene therapy trials are particularly arduous.

## Author Contributions

The authors listed have made substantial, direct and intellectual contributions to the work, and approved it for publication.

## Conflict of Interest Statement

The authors declare that the research was conducted in the absence of any commercial or financial relationships that could be construed as a potential conflict of interest.
